# An Optimized, Slowly Digested Savory Cluster Reduced Postprandial Glucose and Insulin Responses in Healthy Human Subjects

**DOI:** 10.1093/cdn/nzz006

**Published:** 2019-01-17

**Authors:** Thomas M S Wolever, Alexandra L Jenkins, Jun Yang, Mark Nisbet, Jodee Johnson, YiFang Chu, Yang Pan

**Affiliations:** 1 GI Labs, Inc., Toronto, Ontario, Canada; 2 Analytical Sciences, PepsiCo R&D, Plano, TX; 3 Product Development, Barrington, IL; 4 Nutrition Sciences, PepsiCo R&D, Barrington, IL; 5 Nutrition Sciences, PepsiCo R&D, Plano, TX

**Keywords:** snacks, starch, glycemic response, insulinemic response, clinical trial, humans

## Abstract

**Background:**

Slowly digested carbohydrates are perceived as beneficial by some consumers, and various regulatory bodies have published specific criteria defining lower postprandial glycemic response. We developed an optimized savory cluster snack containing slowly digested starch.

**Objective:**

We compared the glucose and insulin responses elicited by the optimized (test-) cluster, a control-cluster, and an available-carbohydrate-matched portion of white bread in healthy individuals. The primary outcome was blood-glucose peak rise.

We tested healthy individuals (*n* = 25) on 3 occasions using a randomized crossover design. On each occasion, the participants provided fasting blood samples and then consumed 1 serving of test-cluster, control-cluster, or white bread. We then measured the participants’ blood-glucose and serum-insulin concentrations over the next 4 h.

**Results:**

The test-cluster elicited a significantly lower blood-glucose peak rise (mean ± SEM: 1.24 ± 0.09 mmol/L) and incremental area under the curve (iAUC; 67 ± 8 mmol × min/L) than the control-cluster (2.27 ± 0.13 mmol/L and 117 ± 10 mmol × min/L, respectively) and white bread (2.27 ± 0.16 mmol/L and 114 ± 9 mmol × min/L, respectively). The serum-insulin peak rise and iAUC elicited by the test-cluster (128 ± 13 pmol/L and 6.10 ± 0.73 nmol × min/L, respectively) and white bread (141 ± 20 pmol/L and 6.47 ± 1.11 nmol × min/L, respectively) were significantly lower than those elicited by the control-cluster (205 ± 26 pmol/L and 9.60 ± 1.31 nmol × min/L, respectively).

**Conclusion:**

The test-cluster elicited lower glucose and insulin responses than the control-cluster. The results support the hypothesis that the carbohydrates in the test-cluster are digested and absorbed slowly in vivo.

## Introduction

Slowly digested carbohydrates elicit low postprandial glycemic responses (PPGRs) (1). Ingredients that reduce PPGRs include viscous dietary fibers (2–4), foods with a low glycemic index (GI) (5, 6), and slowly digested starch (7, 8). Diets containing such ingredients have been shown to assist in weight management (9), and there is some evidence they may reduce perceived exertion during exercise (10), improve cognitive performance (11), and influence substrate oxidation (12). It is therefore of interest to develop and test snacks with a low PPGR.

Both Health Canada and the European Food Safety Authority require that foods carrying a claim related to low PPGRs must not only significantly reduce PPGRs compared with an appropriate control but also not disproportionately increase the insulin response (13–15).

A new process was developed to produce an optimized savory cluster snack in which nuts and whole grains are packed into a hemisphere mold and bound together with flour and soluble fiber,

resulting in a snack approximately 2 cm in diameter. The product is baked using a proprietary low-moisture, low-temperature process to minimize starch gelatinization and reduce the rate ofcarbohydrate digestion. Our objective was to test the hypothesis that the optimized savory cluster (test-cluster) elicits lower postprandial blood-glucose and serum-insulin responses than a control-cluster made from ingredients commonly used in commercially available snacks or bars (oats, peanuts, and corn syrup) and baked using typical processing procedures. Single servings of the test- and control-clusters contain similar amounts of total carbohydrate, protein, fat, and energy. However, because the test-cluster contains ∼30% less available carbohydrate than the control-cluster, we compared the postprandial responses elicited by the test-cluster with those elicited by a portion of white bread with the same available-carbohydrate content as the test-cluster.

## Methods

### Physicochemical property characterization

The moisture content of the test- and control-clusters was determined in triplicate using a TGA-701 Thermogravimetric Analyzer (Leco Corporation). Each cup of the analyzer was loaded with 2–4 g of sample and loaded into the carousel, which was set at 103°C.

Sample texture was measured using a TA.XT2 Texture Analyzer (Stable Micro Systems). Briefly, each sample was compressed by a 45-mm aluminum plate and tested under the following conditions: pretest speed = 1.0 mm/s, test speed = 20.0 mm/s, strain = 75%, and trigger force = 5.0 g. We randomly chose 30 samples of each type of cluster for testing. We recorded the peak force of the samples.

Sample viscosity was measured in singlicate using a Rapid Visco Analyzer 4500 model (Perten Instruments) interfaced with a PC and Thermocline for Windows version 3 software for operation and data management. For viscosity analysis, 3.5 g of ground sample was combined with water to make a mixture with a total mass of 28 g.

We determined the microstructure of the samples by X-ray microtomography using a SkyScan 1172 high-resolution system (SkyScan NV). The cone beam source was set at 40 kV/250 µA for the scanning of hard materials with optimum contrast of void (porosity) and matter (solid). Two-dimensional images were captured using a 16-bit, cooled CCD camera (8000 × 8000 pixels). We obtained a pixel size of 5.938 µm. TNRecon reconstruction software (V1.6.10.4) was used to combine the 2-dimensional, cross-sectional images into a 3-dimensional object. Each 3-dimensional stack contained 1084 virtual sections, each consisting of 1500 × 1500 isotropic voxels with a linear X-ray attenuation coefficient, displayed as gray-scale values calibrated between 0 and 255 in a histogram. CTAn software (Micro Photonics) was used to obtain the total volume of interest, object volume, percentage object volume, structure thickness, and number of closed pores for each sample.

Insoluble dietary fiber, soluble dietary fiber (SDF), and total dietary fiber (TDF) were measured using the AOAC 2011.25 system (16) at Covance Laboratories. Rapidly available glucose (RAG) and slowly available glucose (SAG) were measured as previously described (17).

### Clinical study

**FIGURE 1:**
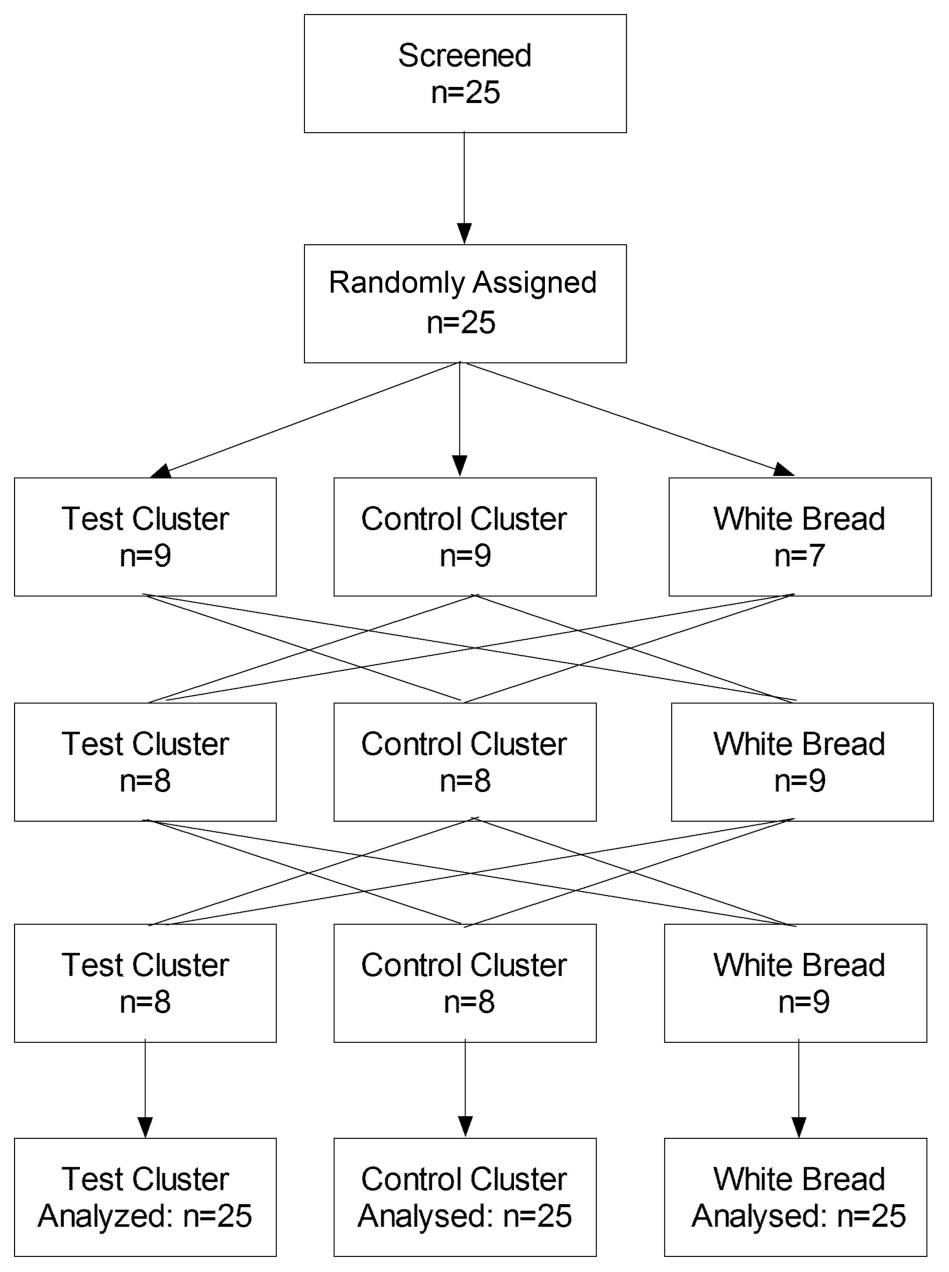
Flow chart of the experimental design.

We conducted a randomized, controlled, crossover study with the aim of having 25 healthy volunteers successfully complete the project (**Figure 1**). The participants were healthy males or nonpregnant females 18–60 y of age with 20 kg/m² < BMI < 35 kg/m² and fasting serum glucose < 7.0 mmol/L (126 mg/dL). As indicated in the protocol, subjects were compensated for their participation. The Western Institutional Review Board approved the study protocol, and all participants provided informed consent before participation. The trial was registered on clinicaltrials.gov as NCT02692144.

Eligible participants, recruited from the pool of subjects who had previously participated in studies at GI Labs, were studied on 3 separate days over a period of 2–4 wk. The interval between successive tests was ≥48 h and ≤2 wk. On each test day, the participants came to GI Labs in the morning after a 10–12-h overnight fast. Participants were asked to maintain stable dietary and activity habits throughout the study period and to refrain from drinking alcohol and to avoid unusual levels of food intake or physical activity for 24 h before each test. If a participant was feeling unwell or did not meet the pretest conditions, that participant's test was rescheduled for another day.

On each test occasion, participants were weighed, and 2 fasting blood samples, 5 min apart, were obtained by finger-prick. Immediately after the second blood sample, participants started to consume a test meal. Participants were asked to consume the entire test meal within 10 min. Further blood samples were obtained for glucose analysis at 10, 20, 30, 40, 50, 60, 90, 120, 180, and 240 min after the first bite was taken. Additional blood was placed into separate vials for insulin analysis at −5, 0, 20, 40, 60, 90, 120, 180, and 240 min. Participants’ hands were warmed with an electric heating pad for 3–5 min before each blood sample. The participants remained seated quietly during the entire 4-h test. After the last blood sample, participants were offered a snack and allowed to leave.

Blood samples for glucose analysis consisted of 2–3 drops of blood collected into 7-mL tubes (Sarsted Inc.) containing potassium oxalate and sodium fluoride; tubes were rotated to mix the blood with the anticoagulant and then placed in a refrigerator until the last blood sample was taken. Each set of 12 samples was bundled together with a rubber band and stored at −20°C until analysis. Glucose analysis was performed within 3 d of sample collection using a model 2300 STAT analyzer (Yellow Springs Instruments). The analytical CV for glucose, calculated from duplicate measures of glucose in all the 0-min samples as CV = 100 × SD/mean, where SD = √(Σ*d*^2^/2*n*), with *d* being the difference between duplicate measures and *n* the number of samples measured in duplicate, was 0.9%.

Blood samples for insulin analysis consisted of 6–8 drops of blood collected into 0.3-mL clot activator containing microvettes (Sarsted Inc.). Tubes were left at room temperature to allow the blood to clot and then centrifuged to remove the serum, which was stored at −20°C before analysis using the Human Insulin EAI Kit (Alpco Diagnostics, catalog #80-INSHU-E10.1). The SD (SD = √(Σ*d*^2^/2*n*), where *d* was the difference between the −5 and 0 min measures, and *n* was the number of 0 min samples) of the insulin concentration measured in the 2 fasting samples (−5 min and 0 min) was 11.0 pmol/L (1.82 μU/mL).

### Test meals

The test meals consisted of 1 serving (56 g) of test-cluster, 1 serving (56 g) of control-cluster, or 47 g of white bread (**Table 1**). We used the same serving size for the test-cluster and the control-cluster because consumers typically choose between products with similar serving sizes and weights as opposed to choosing between products with similar available carbohydrate content. On the other hand, for the white bread, which served as a positive control, we used an amount that contained the same available carbohydrates as the test-cluster. The test-cluster had a RAG:SAG ratio (17) of 2.4, whereas the control-cluster had a RAG:SAG ratio of 10.8 (Table 1). The test-cluster and control-cluster were indiscernible in appearance and had similar organoleptic properties. Each test meal was served with 1 or 2 cups of coffee, tea, or water, and 30 mL of 2% milk if desired. At the first visit, each subject selected the type and volume of drink desired, and this was kept constant for the subsequent 2 visits. The test- and control-clusters were provided in coded packages containing a single serving. The white bread was baked in an automatic bread maker as previously described (18). Each participant received the 3 different test meals in a random order over 3 visits based on a computer-generated schedule.

**TABLE 1:** Nutrient content of the test meals^1^

					Carbohydrate, g					
Test meal	Weight, g	Energy, kcal	Protein, g	Fat, g	Total	Fiber	Sugars	Available^2^	Rapidly available glucose	Slowly available glucose
Test-cluster	56	224	5	12	34	10	1	24	16.2	6.7
Control-cluster	56	250	5	11	35	2	5	33	30.2	2.8
White bread	47	120	4	0.5	25	1	0	24	22.6	1.4

^1^Data were provided by the sponsor.

^2^Calculated as total minus fiber.

The incremental area under the curve (iAUC) of the blood-glucose and serum-insulin responses, ignoring the area below the fasting level, was calculated using the trapezoid rule (19). The peak rise was the maximum concentration of glucose or insulin measured during the 4-h test minus the fasting concentration. The fasting glucose concentration was the mean of the glucose concentrations in the 2 fasting samples.

### Statistical analysis

We wished to have high power to detect a 15% difference in the glucose peak rise. Based on the SD (0.635 mmol/L) of the differences in glucose peak rise in a previous study (20), and using the *t*-distribution to calculate the power, we determined that, with 25 participants, the study had 95% power to detect a 15% difference in glucose peak rise.

Unless otherwise indicated, results are presented as means ± SEM. The primary endpoint was the peak rise in blood-glucose concentration. We compared endpoints using repeated-measures ANOVA, examining the effects of the order and the treatment. The ANOVA was performed on an Excel spreadsheet (version 14.0.7224.5000, Microsoft Corp.) using the linear model (21). After demonstration of significant heterogeneity, individual means were compared using Tukey's test to adjust for multiple comparisons. The moisture content and peak force of the test- and control-clusters were compared by unpaired *t*-test. The criterion for statistical significance was a 2-tailed *P* value of <0.05.

## Results

### Physicochemical properties

The moisture content (mean ± SD) of the test-cluster, 5.0 ± 0.0, was significantly greater than that of the control-cluster, 4.0 ± 0.0 (*P* < 0.05). The peak force of the test-cluster was lower than that of the control-cluster (31.0 ± 6.5 kg compared with 40.0 ± 7.5 kg), although the difference was not statistically significant. The peak viscosity of the test-cluster, 380 cp, was more than twice that of the control-cluster, 127 cp.

**FIGURE 2:**
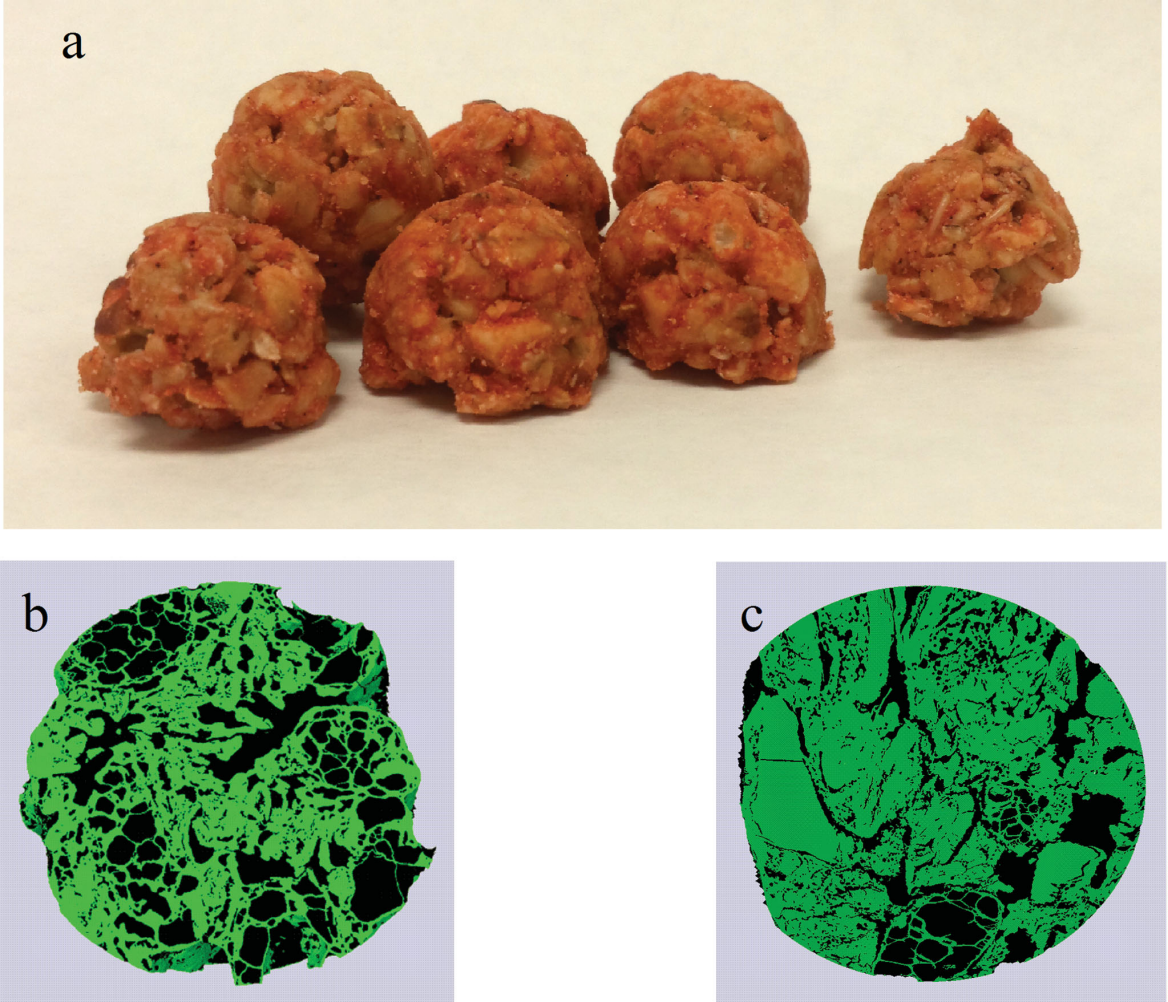
Photograph of (a) test-cluster and 3-dimensional microstructure of (b) the control-cluster and (c) the test-cluster (green color: solid; black color: void space).

The test-clusters and the microstructures of the control- and test-clusters are shown in **Figure 2**. The porosities of the control- and test-clusters were 54.5% and 36.1%, respectively. The reduced porosity of the test-cluster relative to that of the control-cluster is consistent with its slightly harder texture.

The test-cluster contained 1.8, 2.7, and 2.3 times more insoluble dietary fiber, SDF, and TDF, respectively, than the control-cluster. Fifty-eight percent of the TDF in the test-cluster was water-soluble, compared with 49% of that in the control-cluster (**Table 2**).

**TABLE 2:** Low- and high-molecular-weight compound distribution of total dietary fiber in the control- and test-clusters^1^

	Control-cluster		Test-cluster	
	g/100 g	g/test meal	g/100 g	g/test meal
LMWSDF	2.8	1.6	9.0	5.0
SDFP	1.9	1.1	3.9	2.2
IDF	5.1	2.8	9.2	5.2
High-molecular-weight dietary fiber (SDFP + IDF)	7.0	3.9	13.1	7.3
Total soluble dietary fiber (LMWSDF + SDFP)	4.7	2.7	12.9	7.2
Total dietary fiber (LMWSDF + SDFP + IDF)	9.8	5.5	22.1	12.4

^1^IDF, insoluble dietary fiber; LMWSDF, low-molecular-weight soluble dietary fiber; SDFP, soluble dietary fiber precipitable.

### Clinical study

The 25 participants (14 male and 11 female) recruited for the study had a mean ± SD age of 37 ± 11 y and BMI of 25.2 ± 3.0 kg/m², and all 25 completed the study (Figure 1). All participants had a fasting glucose <5.1 mmol/L (92 mg/dL). Twenty-two participants took no prescription medications or supplements. One participant took a daily multivitamin, 1 took 300 mg venlafaxine daily, and 1 took 0.5 mg clonazepam daily.

**FIGURE 3:**
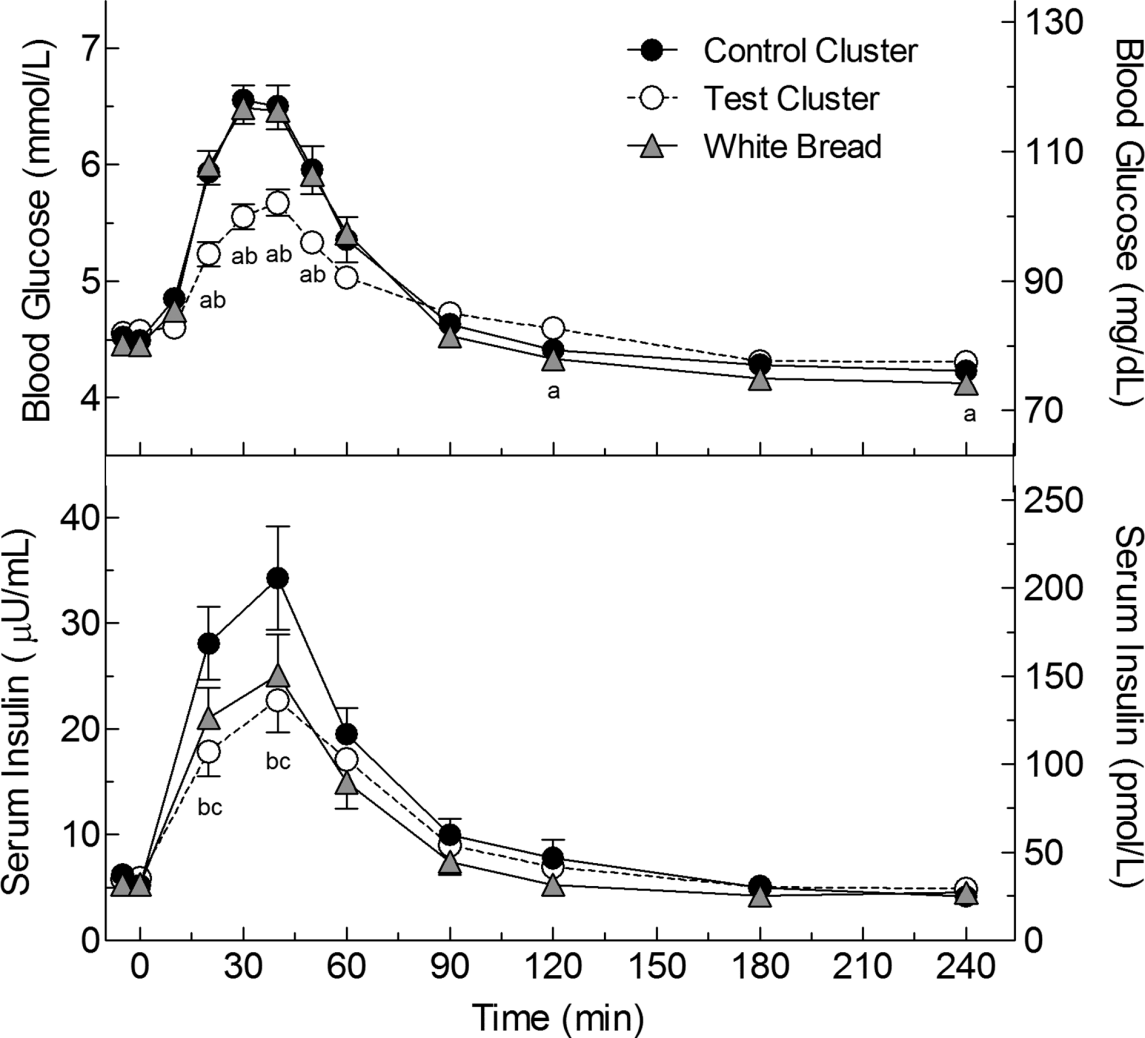
Glucose and insulin responses after the different meals. Points are means ± SEs for *n* = 25 subjects. ^a^Significant difference between test-cluster and white bread (*P* < 0.05 by Tukey's test). ^b^Significant difference between test-cluster and control-cluster (*P* < 0.05 by Tukey's test). ^c^Significant difference between control-cluster and white bread (*P* < 0.05 by Tukey's test).

There was no significant effect of treatment order on blood-glucose or serum-insulin concentration at any time point. The blood-glucose concentration elicited by the test-cluster was significantly lower than that elicited by white bread or the control-cluster at 20, 30, 40, and 50 min and was significantly higher than that elicited by white bread at 120 min and 240 min (**Figure 3**). The serum-insulin concentrations elicited by the test-cluster and white bread at 20 min and 40 min were significantly lower than that elicited by the control-cluster (Figure 3).

The glucose peak rise elicited by the test-cluster was lower than that elicited by the control-cluster (*P* < 0.0001) and lower than that elicited by white bread (*P* < 0.0001; **Table 3**). The insulin peak rise after the test-cluster was similar to that after white bread, but lower than that after the control-cluster (*P* < 0.0001, Table 3). Relative to the control-cluster, the 29 ± 7% reduction in insulin peak rise after the test-cluster was not significantly different (by paired *t*-test) from the 44 ± 4% reduction in glucose peak rise.

**TABLE 3:** Peak rise and incremental areas under the curve for glucose and insulin^1^

	Peak rise	iAUC 0–2 h	iAUC 2–4 h	iAUC 0–4 h
Glucose	mmol/L		mmol × min/L	
Test-cluster	1.24 ± 0.09^b^	62 ± 7^b^	4.8 ± 1.9	67 ± 8^b^
Control-cluster	2.27 ± 0.13^a^	112 ± 10^a^	4.8 ± 1.7	117 ± 10^a^
White bread	2.27 ± 0.16^a^	110 ± 9^a^	3.5 ± 2.1	114 ± 9^a^
Insulin	pmol/L		nmol × min/L	
Test-cluster	128 ± 13^b^	5.88 ± 0.71^b^	0.22 ± 0.07	6.10 ± 0.73^b^
Control-cluster	205 ± 26^a^	9.23 ± 1.25^a^	0.37 ± 0.13	9.60 ± 1.31^a^
White bread	141 ± 20^b^	6.30 ± 1.06^b^	0.17 ± 0.06	6.47 ± 1.11^b^

^1^Results are given as means ± SEMs for *n* = 25 subjects. Means within a column with a different superscript differ significantly (Tukey's test, *P* < 0.05). iAUC, incremental area under the curve.

The glucose iAUC 0–2 h and iAUC 0–4 h elicited by the test-cluster were significantly lower than those after both the control-cluster and white bread (all *P* < 0.0001; Table 3). The insulin iAUC 0–2 h and iAUC 0–4 h after the test-cluster were significantly lower than those after the control-cluster but similar to white bread (Table 3). Relative to control-cluster, the percentage reduction in insulin iAUC after the test-cluster, 31 ± 6%, was not significantly different from the 42 ± 5% reduction in glucose iAUC.

## Discussion

We hypothesized that the test-cluster would elicit lower glucose and insulin responses than the control-cluster because it would be digested more slowly. However, a single serving of the test-cluster contained 27% less available carbohydrate (avCHO) than a serving of the control-cluster (24 g compared with 33 g), which would reduce its relative glycemic and insulinemic impacts. As avCHO intake increases, glycemic response, measured as 0–2-h iAUC, increases in a nonlinear fashion (18, 22). The 0–2-h iAUC elicited by a food, relative to that elicited by 50 g of glucose, termed the relative glycemic response, can be estimated from the following equation:

(1) \begin{equation*} {\rm{Relative}}\,{\rm{glycemic}}\,{\rm{response}} = 1.49 \times {\rm{GI}} \times (1 - {{\rm{e}}^{ - 0.0222 \,\, {\rm{g}}}})\end{equation*}

where g is the weight (grams) of avCHO consumed, and GI is the GI of the food (23). Equation *1* indicates that the relative glycemic response for 33 g glucose (GI = 100) is 77.4, whereas that for 24 g glucose is 61.5. Therefore, 24 g of avCHO would be expected to elicit a 0–2-h iAUC 79.5% of that elicited by 33 g avCHO (a 20.5% reduction). The results showed that the mean 0–2-h iAUC elicited by the test-cluster was 55% of that elicited by the control-cluster (a 45% reduction). The difference in avCHO only accounts for about 46% of the observed reduction in glycemic response. Hence, the additional observed reduction in glycemic response is likely due to the avCHOs in the test-cluster being digested more slowly than those in the control-cluster. The test-cluster contained less RAG than the control-cluster, 16.2 compared with 30.2 g; based on Equation *1*, 16.2 g glucose would elicit a 55% lower glycemic response than 30.2 g glucose; this difference agrees with the observed 55% reduction in 0–2-h iAUC.

The glycemic response curve elicited by the test-cluster was flatter than that elicited by the other test meals, with a lower peak and a more sustained elevation of glucose 2–4 h after eating, a pattern thought to be associated with slower starch digestion in vivo. The latter is supported by the lower amount of RAG and higher amount of SAG in the test-cluster. The physicochemical properties of dietary carbohydrates influence their rates of digestion. Factors that may be associated with rapid digestion include factors such as a more porous food matrix structure or a greater degree of starch gelatinization (24). The test-clusters had significantly lower porosity than the control-clusters, which may have reduced the surface area of starch readily accessible to pancreatic amylase during digestion. The greater viscosity of the test-clusters suggests that they contained less gelatinized starch. However, the higher viscosity of the test-cluster could also have resulted, at least in part, from the higher amount of high-molecular-weight (MW) SDF it contained compared to the control-cluster. The ability of soluble fiber to reduce glycemic responses is directly related to the amount consumed and its MW (25). Although we did not measure the MW of the fiber present in the test-cluster, it contained about 2.5 times more high-MW soluble fiber per gram avCHO than the control-cluster (0.16 g/g compared with 0.06 g/g). It has been shown that granola bars made with oats and containing ratios of high-MW oat β-glucan to avCHO of 0.16 and 0.11 g/g, respectively, elicit 25% and 19% reductions in iAUC compared to control granola bars made with wheat (25).

The test-cluster contained the same amount of avCHO as white bread, but more fat (12 compared with 0.5 g), a factor that may have contributed to its significantly lower iAUC 0–2 h and significantly higher blood glucose 2 and 4 h after eating (26). The dose–response effect of fat on blood glucose 2–4 h after eating is not known. However, adding 0, 5, 10, 20, and 40 g of fat (margarine) to a portion of white bread containing 50 g avCHO (27) reduced mean glucose 0–2 h iAUC in a nonlinear fashion as follows:

(2) \begin{equation*}{\rm{iAUC}} = (47 \times {{\rm{e}}^{ - 0.0522\, {\rm{g}}}}) + 119.\end{equation*}

The test-cluster contained 25 g of fat per 50 g of avCHO; inserting that value into Equation *2* results in an estimated iAUC of 132, which is 79% of the iAUC for 0 g of fat. The 0–2-h iAUC elicited by the test-cluster was 55% of that after white bread (a 45% reduction); dividing 55% by 0.79 results in a value of 70% (a 30% reduction), which represents the glycemic impact of the test-cluster relative to white bread, adjusted for the difference in fat. Thus, we estimate that one-third of the 45% reduction in 0–2 h iAUC after the test-cluster compared to white bread was due to its higher fat content and two-thirds to slower carbohydrate absorption.

Our results showed that the insulin 0–2-h iAUC elicited by the test-cluster was 31% lower than that elicited by the control-cluster, and not significantly different from the 42% reduction in the glucose response. Thus, our results comply with the requirement from the European Food Safety Authority and Health Canada that foods claiming to elicit a reduced glycemic response not disproportionately increase the insulin response. However, compared to white bread, the control-cluster elicited a 47% higher insulin response in the face of a virtually identical glycemic response, and the test-cluster elicited a similar insulin response in the face of a significantly lower glycemic response. Fatty acids are known to potentiate the ability of glucose to stimulate insulin secretion from human pancreatic islets in vitro (28). In addition, fat consumption increases postprandial insulin secretion in vivo by stimulating the secretion of glucose-dependent insulinotropic polypeptide (29). Thus, the higher fat content of the test- and control-clusters may explain why the insulin responses they elicited, relative to that elicited by white bread, were higher than their relative glucose responses. However, the insulin response of the test-cluster was not disproportionately increased compared to that of the control-cluster, because both clusters contained similar amounts of fat.

Approximately 42% of the available carbohydrates in the control-cluster came from corn syrup, compared with virtually none in the test-cluster. This could have contributed to the higher glycemic impact of the control-cluster, because corn syrup consists of dextrins and the sugars maltose and glucose (the source of almost all the sugars in the control-cluster). Dextrins and maltose have a GI equivalent to that of glucose (i.e., 100) and higher than that of most refined starchy foods (30) and elicit postprandial responses characterized by a rapid rise and early peak of blood glucose followed by a rapid fall and undershoot. The pattern of response for the test-cluster was more consistent with that expected for slowly digested starch and therefore presumably reflects the higher RAG:SAG ratio in the test-cluster than in the control-cluster.

We conclude that, compared with the control-cluster, the test-cluster elicited a reduced postprandial glycemic response without a disproportionate increase in the postprandial insulin response. Our results support the hypothesis that the carbohydrates in the test-cluster are digested slowly in vivo.

## Notes

Funding for the study was provided by PepsiCo, Inc.

Author disclosures: TMSW and ALJ are part owners and employees of GI Labs, Inc., a contract research organization. Neither they nor GI Labs have any financial interest in any intellectual property that has been or may be developed from the results reported here; nor do they own shares in PepsiCo, Inc., or any of its subsidiaries. JY, JJ, MN, YP, and YC are employees of PepsiCo, Inc. The views expressed in this manuscript are those of the authors and do not necessarily reflect the position or policy of PepsiCo, Inc.

Abbreviations used: iAUC, incremental area under the curve; MW, molecular weight; PPGR, postprandial glycemic response; RAG, rapidly available glucose; SAG, slowly available glucose; SDF, soluble dietary fiber; TDF, total dietary fiber.
